# A local evaluation of the individual state‐space to scale up Bayesian spatial capture–recapture

**DOI:** 10.1002/ece3.4751

**Published:** 2018-12-18

**Authors:** Cyril Milleret, Pierre Dupont, Christophe Bonenfant, Henrik Brøseth, Øystein Flagstad, Chris Sutherland, Richard Bischof

**Affiliations:** ^1^ Faculty of Environmental Sciences and Natural Resource Management Norwegian University of Life Sciences Ås Norway; ^2^ Centre National de la Recherche Scientifique (CNRS), Unité Mixte de Recherche (UMR) 5558, Laboratoire de Biométrie et Biologie Évolutive Université Lyon 1 Villeurbanne France; ^3^ Norwegian Institute for Nature Research Trondheim Norway; ^4^ Department of Environmental Conservation University of Massachusetts Amherst Massachusetts USA

**Keywords:** computation speed, local evaluation of the state-space, spatial capture–recapture, wolverines

## Abstract

Spatial capture–recapture models (SCR) are used to estimate animal density and to investigate a range of problems in spatial ecology that cannot be addressed with traditional nonspatial methods. Bayesian approaches in particular offer tremendous flexibility for SCR modeling. Increasingly, SCR data are being collected over very large spatial extents making analysis computational intensive, sometimes prohibitively so. To mitigate the computational burden of large‐scale SCR models, we developed an improved formulation of the Bayesian SCR model that uses local evaluation of the individual state‐space (LESS). Based on prior knowledge about a species’ home range size, we created square evaluation windows that restrict the spatial domain in which an individual's detection probability (detector window) and activity center location (AC window) are estimated. We used simulations and empirical data analyses to assess the performance and bias of SCR with LESS. LESS produced unbiased estimates of SCR parameters when the AC window width was ≥5σ (*σ*: the scale parameter of the half‐normal detection function), and when the detector window extended beyond the edge of the AC window by 2*σ*. Importantly, LESS considerably decreased the computation time needed for fitting SCR models. In our simulations, LESS increased the computation speed of SCR models up to 57‐fold. We demonstrate the power of this new approach by mapping the density of an elusive large carnivore—the wolverine (*Gulo gulo*)—with an unprecedented resolution and across the species’ entire range in Norway (> 200,000 km^2^). Our approach helps overcome a major computational obstacle to population and landscape‐level SCR analyses. The LESS implementation in a Bayesian framework makes the customization and fitting of SCR accessible for practitioners working at scales that are relevant for conservation and management.

## 1 INTRODUCTION

Spatial capture–recapture (SCR) models are now routinely used in ecological studies to estimate density of animal populations using encounter history data from recognizable individuals (Borchers & Efford, 2008; Efford, 2004; Royle & Young, 2008). More recently, extensions of SCR have been used to investigate many aspects of spatial ecology (Royle, Fuller, & Sutherland, 2018), including dispersal and survival (Ergon & Gardner, 2014; Schaub & Royle, 2014), landscape connectivity (Sutherland, Fuller, & Royle, 2015), habitat fragmentation (Bischof, Steyaert, & Kindberg, 2017), landscape conservation management (Morin, Fuller, Royle, & Sutherland, 2017), and epidemiology (Muneza et al., 2017). The basic principle of SCR models is the use of spatial patterns of individual detection/non‐detections to estimate detection probability as a function of the distance from individual activity centers. The activity center positions are themselves latent variables which are estimated using a spatial point process model.

A growing number of studies employ noninvasive methods, such as camera trapping and noninvasive genetic sampling, for monitoring wildlife. These approaches allow the collection of data suitable for SCR analysis at the level of populations and landscapes (Bischof, Brøseth, & Gimenez, 2016). The greatest hurdle to scaling up inferences to increasingly large spatial domains is the computational burden associated with fitting spatially explicit model and individual‐based model to data collected over a large spatial extent. Point process models offer flexibility and are an important strength of the state‐space formulation of SCR; however, they can quickly become computationally intensive or even prohibitive with increasing size of the spatial domain (Milleret, Dupont, et al., 2018).

Here, we motivate the need for improved computational efficiency of SCR models using our own challenge attempting to estimate population densities of three large carnivores species (wolves *Canis lupus*, Brown bears *Ursus arctos*, and wolverine *Gulo gulo*) using noninvasive genetic sampling (NGS) data collected across their entire range in two countries (Norway and Sweden). For example, fitting a classical formulation of the SCR model to wolverines NGS data collected over its entire range in Norway using a Bayesian analysis (Royle & Young, 2008) was not possible on a standard desktop computer due to memory constraints.

A typical SCR model assumes that the study area is large enough to contain several individuals representing the population of interest. This implies that, during the study period, individuals occur within smaller subsets (i.e., windows) of the study area defined by the home range size. Therefore, the spatial pattern of observations of known individuals will be restricted to areas defined by their space use and ranging behavior over the study duration. This is explicitly accounted for in SCR by modeling detection probability as a decreasing function of the distance between an individual's activity center (AC) and a detector (e.g., device: camera and hair snare; observer: NGS transects). As the size of the study area (hereafter named spatial domain) increases relative to the species home range size, the fraction of the spatial domain not used by a given individual (i.e., where its detection probability is effectively 0) increases. In the current SCR formulation, all detectors are included in the likelihood optimization when fitting models, which is both inefficient and useless because null detection probability is non‐informative. Therefore, removing likelihood calculations for an individual in parts of the spatial domain where its detection probability is close to 0, which we refer to as “local evaluation,” should result in improved computational efficiency without affecting SCR parameter estimates.

Prior information on the study species’ ranging behavior can be used to inform the spatial scale of the localized likelihood evaluation, which essentially produces individual level state‐spaces that are nested within the entire state‐space of SCR models. In other words, local evaluation restricts the spatial domain available to each individual. While the local evaluation approach is implemented in oSCR (Sutherland, Royle, & Linden, 2017), a package for fitting SCR models using integrated likelihood, the approach has not been implemented for more time‐intensive and widely applied Bayesian analyses of SCR models. In addition, frequentist implementation of SCR does not offer the same flexibility and accessibility to build complex models that have made Bayesian SCR models popular with many ecologists.

**Figure 1:**
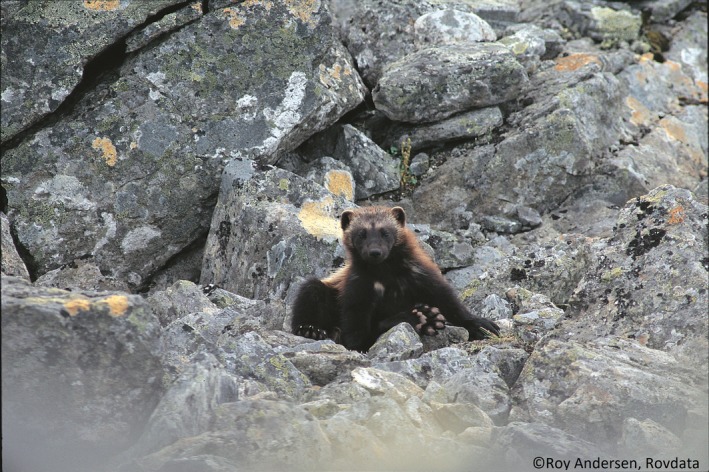
Photography of a wolverine (*Gulo gulo*) © Roy Andersen, Rovdata

Here, we describe an approach for local evaluation of the individual state‐space (LESS) for computationally efficient fitting of large‐scale SCR models in a Bayesian framework. We use simulations to assess the performance of the LESS approach, both in terms of computation speed and the performance of the estimators. We then use the LESS approach to estimate wolverine density throughout Norway using non‐invasive genetic sampling data from the national monitoring program (Figure 1) on a standard desktop computer, a task that we were unable to execute using SCR without LESS. We provide example R codes and functions for implementing our approach in JAGS (Plummer, 2003).

## 2 MATERIAL AND METHODS

### 2.1 Bayesian formulation of SCR models

In SCR, the spatial locations of detections and non‐detections at a set of detectors are used to estimate the latent locations of individual activity centers (ACs). SCR models are hierarchical state‐space models that combine a spatial point process model describing the spatial distribution of individual ACs, and an observation model, describing the relationship between individual detection probability and distance to its AC. The classical half‐normal detection model assumes that the probability *p* of detecting individual *i *at detector *j* decreases with distance between the detector and the AC (*D_ij_*):

(1) $$p_{\mathrm{ij}}=p_{0}\cdot \exp\left(\frac{-D_{\mathrm{ij}}^{2}}{2\sigma^{2}}\right)$$

where *p_0_* and *σ* are the magnitude and scale parameters, respectively. While *p_0_* represents the detection probability at the location of the AC, *σ* is directly related to the width of the utilization distribution, that is, a function of home range size (Royle, Chandler, Sollmann, & Gardner, 2013). More generally, *σ* is related to the extent of space used over the period of study (see Royle et al. (2013) to relate *σ* to area used).

Standard SCR models assume that location of ACs (*s_i_*) is uniformly distributed across the spatial domain (*S*) which is modeled as a homogeneous point process:

(2) $$s_{i}∼\text{Uniform}\left(S\right)$$

In order to constrain the location of ACs within suitable habitat (e.g., irregularly shaped study area), we used the “ones tricks” (Chandler, 2015; Meredith, 2016; Spiegelhalter, Thomas, Best, & Lunn, 2003). This trick consists of determining whether an individual AC was located within suitable habitat or not *pOK_i_* (1/0) and rejecting the proposed AC location if *pOK_i_* = 0 using:

(3) $$OK_{i}∼\text{Bernoulli(}pOK_{i}\text{)}$$

where OK is a set of ones.

Bayesian analysis by Markov Chain Monte Carlo (MCMC) with data augmentation can be used to analyze SCR models (Royle, Dorazio, & Link, 2007; Royle, Karanth, Gopalaswamy, & Kumar, 2009). Data augmentation involves (a) augmentation of the dataset with an arbitrary (but sufficiently large) number of individuals that were never detected, and (b) use of a latent binary variable *z_i_* reflecting the realization of the inclusion probability *ψ*, which is an estimate of the proportion of individuals truly present in the population. Estimated abundance ($\hat{N}$) is then derived by summing over the inclusion vector *z*, and density *d* is derived by dividing $\hat{N}$ by the area of *S.*

### 2.2 Local evaluation of the state‐space

**Figure 2:**
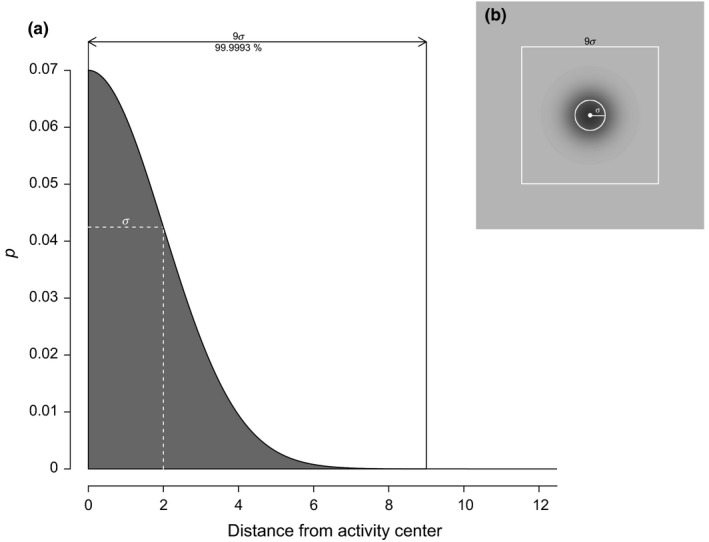
(a) Illustration of the half‐normal detection function (Eq. ((1)) representing a declining detection probability (*p*) of an individual with increasing distance from its activity center (AC) in spatial capture–recapture models. Values for *σ* and *p_0_* were set to 2 and 0.07, respectively. A restriction window with a width of 9*σ* centered on the AC covers 99.9993% of the total range of *p*. (b) Spatial illustration of the detection probability distribution of an individual using the half‐normal detection function. Black shading illustrates the increasing detection probability of an individual around its AC (white dot). The white circle corresponds to a circular area with a radius equal to *σ*. The white square represents a restriction window with a width of 9*σ* centered on the AC

Local evaluation involves restricting the spatial domain over which detection probabilities are estimated and activity center locations are assumed to occur. To restrict the spatial domain of each individual, we used “evaluation windows” centered on the centroid of individual detection locations. Individual windows should be large enough to ensure all plausible activity center locations and to ensure that the detection function falls to approximately zero. At the same time, the evaluation window should be small enough to remove most of the unnecessary computation in large parts of the spatial domain where *p* = 0 or where AC locations are not likely (Figure 2). Because of the state‐space formulation of SCR models, we refer to this approach as the local evaluation of the state‐space (LESS).

**Figure 3:**
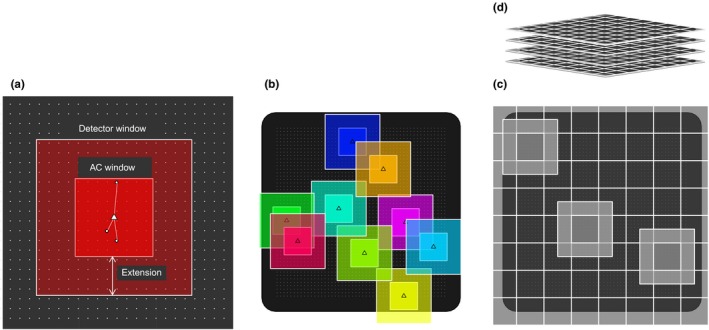
Illustration of the local evaluation of the individual state‐space (LESS) procedure in spatial capture–recapture (SCR). SCR with LESS involves using evaluation windows to restrict the area of the spatial domain used by each individual. The black area and small white dots represent the spatial domain and detectors, respectively. (a) Example of the placement of the activity center (AC) evaluation window (inner dark red square) and detector (outer paler red square) evaluation window centered on the centroid (triangle) of all detections of one individual. “Extension” represents the distance by which the detector window extends beyond the edge of the AC window. (b) Darker inner square evaluation windows delineate the areas within which ACs are located and lighter outer evaluation windows delineate the additional areas within which detections are considered. (c) A grid of AC and associated detector evaluation windows is established to accommodate a layer of augmented individuals. AC windows and associated detector window are shown in lighter shading for three example individuals. (d) Additional layers of AC and detector windows grids are superimposed to reach a desired level of augmentation

The local evaluation of the state‐space was performed using an inner square evaluation window assigned to each individual to restrict the placement of their AC (hereafter the AC evaluation window) and a larger one to restrict the window within which their associated detection can occur (hereafter the detector evaluation window; Figure 3a). For each detected individual, we centered the AC and detector evaluation windows on the centroid of all its detections (Figure 3a–b).

In contrast to the frequentist local evaluation approach implemented by Sutherland et al. (2017), Bayesian formulation of SCR involves data augmentation (Royle et al., 2007, 2009). Therefore, it is necessary to define AC and detector evaluation windows for the unobserved (augmented) individuals. To do so, we juxtaposed AC evaluation windows of augmented individuals over the full extent of the study area (Figure 3c), thus generating a constant density of augmented ACs within the spatial domain. As a result, the width of the AC window relative to the size of the study area dictates the number of augmented individuals. If a larger number of augmented individuals is needed, it is possible to superimpose layers of juxtaposed squared windows (Figure 3d). Depending on their location, AC windows of both detected and augmented individuals may include non‐suitable habitat (i.e., rejection of AC location if in non‐suitable habitat; Figure 3b–c). In such cases, the size of the area available to locate ACs differs among individuals. We therefore defined the inclusion parameter *ψ* for each individual *i* as a function of the proportion of suitable habitat (*prop.habitat*) within their AC window to obtain unbiased density estimates:

(4) $$\psi_{i}=1-{\left(1-\psi_{0}\right)}^{prop.habitat_{i}}$$

Where

(5) $$\psi_{0}∼\text{Uniform}\left(0,1\right)$$

**Figure 4:**
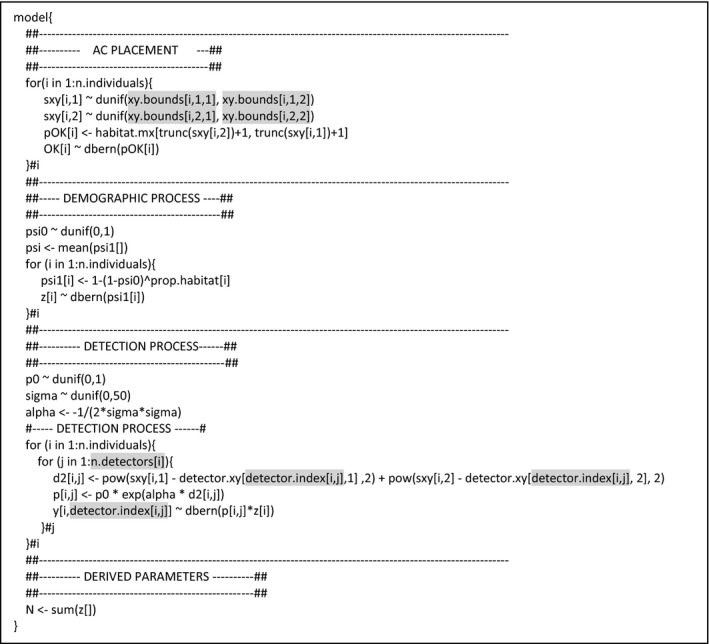
JAGS model specification for the local evaluation of the individual state‐space. The indexing allowing the local evaluation is highlighted in light gray. *xy.bounds* defines the AC evaluation window with an array of *x* and *y* coordinates for each individual. *n.detectors* is a vector of the number of detectors within the detector evaluation window associated with each individual. *detector.index* is a matrix representing the detector indexes included within the detector evaluation window of each individual. In practice, such local evaluation of the individual state‐space requires constraining of: (1) the area within which *s_i_*, the individual AC, can be located, and (2) the detectors used to estimate the detection curve from the observed pattern of detections/non‐detections (Figures 2 and 3). In addition to reducing the number of detectors where individual detections are considered possible, the local evaluation approach also reduces the domain over which individual ACs are likely to occur, further increasing computational efficiency

The formulation of a Bayesian SCR model with LESS relies on proper indexing to specify the extent of AC and detector windows (Figure 4). R code for generating the indexes necessary to implement a SCR model with a LESS is provided in the Supporting Information Appendix S1 and S2.

### 2.3 Simulation scenarios

#### 2.3.1 Population and survey characteristics

We used a 50 × 50 detector array (2,500 detectors located one distance unit (du) apart from each other). The spatial domain was defined as the area including the detectors and a buffer of 4 du* (*2*σ*, see below). We simulated two different population sizes (50 and 100 individuals) and assumed demographic closure (i.e., no recruitment emigration/immigration or death). Individual AC locations were drawn as a uniform random sample from the spatial domain. Following the recommendation by (Sun, Fuller, & Royle, 2014) for the choice of detector spacing relative to *σ*, we simulated binary detection data *y* using Eq. ((6)) with *σ* = 2, and *p_0_* = 0.07 leading to an average detection rate of 66% (range: 57%–78%) of alive individuals (N):

(6) $$y_{\mathrm{ij}}∼\text{Bernoulli}\left(p_{0}.\exp\left(\frac{-D_{i,j}^{2}}{2\sigma^{2}}\right)\right)$$

#### 2.3.2 Local evaluation of the state‐space

**Figure 5:**
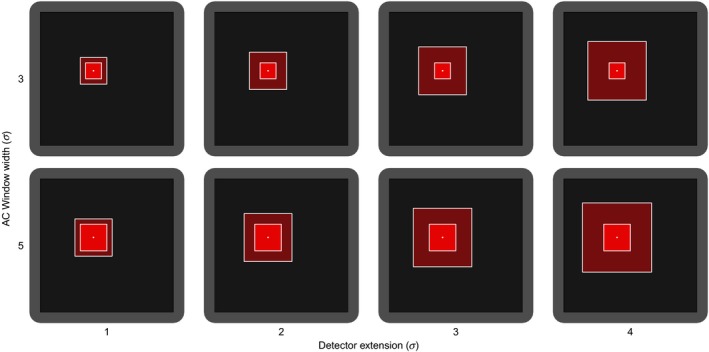
Illustration of the different AC (in rows) and detector (in columns) evaluation windows widths used in the simulations to test the local evaluation of the individual state‐space (LESS) in SCR model. The darker and lighter black areas represent the detector and buffer areas of the spatial domain. The darker and lighter red square represents the AC and detector evaluation windows, respectively. All values are expressed relative to σ. See Figure 3 for a description of the windows

We investigated the performance (see below) of SCR with LESS using different AC and detector evaluation windows widths. We used 3*σ* (6 du) and 5*σ* (10 du) as the width of the AC window (Figure 5). We then sequentially increased the width of the detector evaluation window, so that the detector window extended beyond the edge of the AC window by 1, 2, 3, and 4 times *σ*. This resulted in detector windows of different width according to the AC window chosen (Figure 5). We adjusted the number of layers of AC windows (corresponding to augmented individuals) to obtain >4× number of simulated individuals.

### 2.4 Evaluation of model performance

#### 2.4.1 SCR key parameters

For reference, we fitted an SCR model without local evaluation. We then ran 100 simulations of each scenario and calculated the relative bias $\left(\text{RB =}\;\frac{1}{\theta n}\sum_{i=1}^{n}\left({\hat{\theta }}_{i}-\theta \right)\right)$ and the precision of $\hat{N}$, $\hat{\sigma }$ and ${\hat{p}}_{0}$ for each simulation scenario using the coefficient of variation $\left(\text{CV =}\;\frac{\text{SD(}\hat{\theta }\text{)}}{\hat{\theta }}\times 100\right)$ (Walther & Moore, 2005), where *n* is the number of iterations, *SD* is the standard deviation, θ is the true parameter value, and $\hat{\theta }$ the estimate of the parameter obtained from MCMC. In addition, we calculated the 95% credible interval coverage as the percentage of simulations where the 95% credible interval contained the true parameter value. We also recorded the computing time for each model fit (i.e., time necessary to run three MCMC chains in series on a single computer core). Because we used cores with different characteristics to run the different SCR models, computing times reported only serve as a crude estimate of the time used for model fitting.

#### 2.4.2 Density predictions

One of the main advantage of SCR methods is to derive spatially explicit estimates of density. We therefore quantified the deviation between the simulated and realized density maps produced using SCR (Milleret, Dupont, et al., 2018) models with and without LESS. We constructed true density maps by applying Eq. ((1)) (excluding *p_0_*) to the true simulated AC locations of individuals and summing space use across all individuals at the habitat grid cell level (1 × 1 du). To derive the predicted average space used, we used the uncertainty around the estimates in the posterior samples (location of ACs and *σ*) obtained from the Bayesian Markov chain Monte Carlo (MCMC) and summed predicted average space used of all individuals that were considered as part the population (*z* = 1). To compare the deviation between the shape of true and predicted density maps, we scaled both maps to sum to 1 and calculated mean relative error (MRE) in density as:

(7) $$\text{MRE =}\;∥\frac{D_{j}}{\sum_{i}^{h}{\hat{D}}_{j}}-\frac{D_{j}}{\sum_{i}^{h}D_{j}}∥$$

where ${\hat{D}}_{j}$ is the predicted and *D_j_* the simulated density at cell *j* in a habitat raster consisting of *h* cells. We excluded the buffer area when calculating MRE. We then summarized the average MRE across all simulations for any given scenario.

### 2.5 Application to the wolverine data

We fitted an SCR model with LESS to NGS data from the national monitoring program of wolverines in Norway (see description in Flagstad et al. (2004), Brøseth, Flagstad, Wärdig, Johansson, & Ellegren (2010) and Bischof, Gregersen, Brøseth, Ellegren, & Flagstad (2016)) to illustrate the local evaluation approach. The data consisted of 453 detections from 196 individually identified female wolverines collected using scat‐based noninvasive genetic sampling between January and May 2012. We used a grid with a 2 km resolution and only retained as detectors those grid cells that were searched (i.e., search‐encounter sampling; Russell et al., 2012) by the Norwegian Nature Inspectorate, which resulted in 17,266 detectors, covering a total area of approximately 70,000 km^2^. Individual detections at any given detector were treated as a binary variable (Milleret, Dupont, et al., 2018).

We used AC evaluation windows of 5*σ* (the largest LESS restriction used in our simulation, *σ*≈ 6 km for females wolverines (Milleret, Dupont, et al., 2018)) and detector evaluation windows that extended by 2*σ* beyond the edges of the AC windows. The goal of the empirical analysis was to put the LESS approach to the test on a large‐scale SCR problem, and we used a simple SCR model for this task (as described in section 2.1). Comprehensive and reliable density estimation of wolverine density would require a model that, among other things, accounts for heterogeneity in density and detectability, which is beyond the scope of the analysis presented here. To avoid misinterpretation of density and abundance estimates from our simplified analysis as actionable results with respect to population management, we do not provide absolute estimates, only report computation time and a map of relative density, to be interpreted with caution.

### 2.6 Model fitting

We fitted Bayesian SCR models using Markov chain Monte Carlo (MCMC) with JAGS (Plummer, 2003) and *rjags* (Plummer, 2016) in R version 3.3.3 (R Core Team, 2017). After an adaptive phase of 1,000 iterations, we ran 3,000 iterations of three chains thinned by three. We considered models as converged when the Gelman‐Rubin statistics (*gelman.diag* function in coda package (Plummer, Best, Cowles, & Vines, 2006)) (Brooks & Gelman, 1998; Gelman & Rubin, 1992) was ≤1.1 and after visually inspecting trace plots for all monitored parameters. R and JAGS code for the different SCR models and simulations used are provided in Supporting Information Appendix S1 and S2, and list of priors used in Supporting Information Appendix S3.

## 3 RESULTS

### 3.1 Simulations

**Figure 6:**
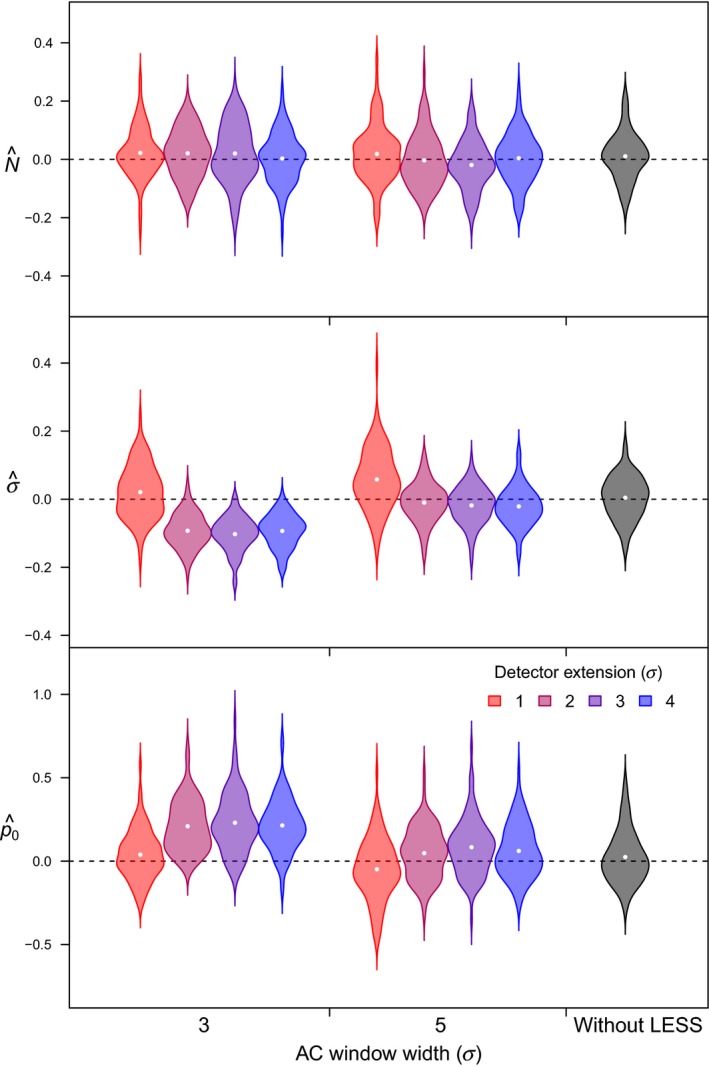
Relative bias of spatial capture–recapture (SCR) model parameters with and without local evaluation of the individual state‐space (LESS) for a simulated population size of 100 individuals. Relative bias plotted against the different widths of the AC evaluation windows and different detector extensions (i.e. the distance by which the detector window extends beyond the edges of the AC window, see Figure 5). AC window width and detector window extension are expressed relative to σ. Violins show kernel density (dots: mean) of the relative bias computed from the posterior distribution of three key SCR parameters ($\hat{N}$, $\hat{\sigma }$, and ${\hat{p}}_{0}$) based on 100 simulations

**Figure 7:**
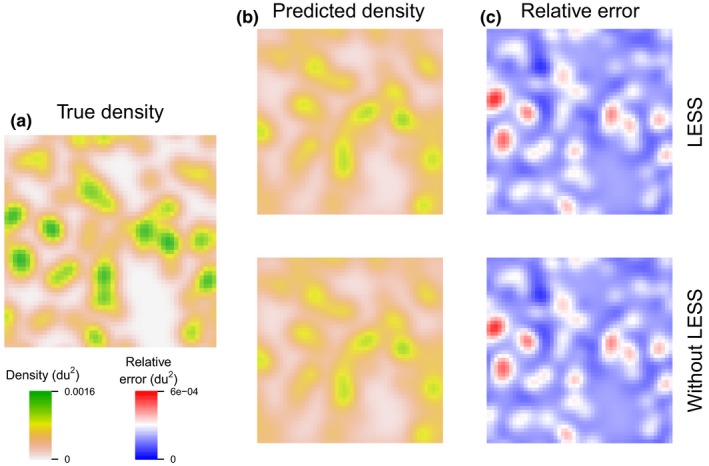
Simulation‐based comparison of true and SCR predicted densities. (a) Example of a true density (du^2^) from a simulated dataset (*N* = 100). (b) Predicted density maps obtained with a SCR model with and without local evaluation of the individual state‐space (LESS). The local evaluation was performed using an AC evaluation window of 10*σ* with a 2*σ *extension of detector windows beyond the edges of AC windows. (c) Relative error between true (a) and predicted (b) density with a SCR model with and without LESS. The SCR model with LESS returns similar density surfaces compared to the SCR model without LESS. Density maps are shown excluding the buffer of the spatial domain

All 1800 models (with and without LESS) reached convergence after 3,000 iterations. Relative bias of SCR models with LESS generally decreased with increasing width of the AC evaluation window (Figure 6, Table 1). When using a detector evaluation window with the smallest extension beyond the AC evaluation window (1*σ*), $\hat{N}$ and $\hat{\sigma }$ tended to be slightly overestimated and ${\hat{p}}_{0}$ underestimated, regardless of the width of the AC window (Figure 6, Table 1). However, as the extension increased (>1*σ*), $\hat{\sigma }$ tended to be underestimated and ${\hat{p}}_{0}$ overestimated. Regardless of the scenario (*N* = 50 or 100), relative bias in $\hat{N}$ (<0.005), $\hat{\sigma }$ (≈−0.01), and ${\hat{p}}_{0}$ (<0.1) were lowest for an AC window that was equal to 5σ (10 du*)* and when detector evaluation window extended 2*σ* beyond the edges of AC window (width of the detector window = 18 du*)*. MRE between true and predicted density surfaces was similar for SCR models with and without LESS (Table 1, Figure 7). With our survey and population characteristics, fitting SCR model with LESS (AC region = 5*σ*, extension between the detector and AC regions = 2*σ*) was between 37–57 times faster than fitting SCR models without LESS (Table 1).

**Table 1:** Average relative bias, coefficient of variation, coverage of key parameters ($\hat{N}$, $\hat{\sigma }$, ${\hat{p}}_{0}$), and average running time of SCR models with and without local evaluation of the state‐space (LESS) from the simulation study

AC windows width	3				5				
detector window extension	1	2	3	4	1	2	3	4	Without LESS
*N* = 100									
$\hat{N}$									
RB	0.02	0.02	0.02	0	0.02	0	−0.02	0	0.01
CV	8.9	8.62	8.65	8.66	9.02	8.68	8.54	8.69	8.86
Coverage	0.94	0.95	0.89	0.96	0.91	0.94	0.92	0.93	0.94
$\hat{\sigma }$									
RB	0.02	−0.09	−0.1	−0.09	0.06	−0.01	−0.02	−0.02	0
CV	7.23	5.4	5.19	5.18	7.56	6.19	5.97	5.99	6.46
Coverage	0.93	0.57	0.47	0.59	0.86	0.94	0.95	0.94	0.94
${\hat{p}}_{0}$									
RB	0.04	0.21	0.23	0.21	−0.05	0.05	0.08	0.06	0.02
CV	16.2	15.19	15.05	15.05	17.62	16.26	15.89	16.05	16.61
Coverage	0.97	0.83	0.76	0.83	0.9	0.97	0.92	0.96	0.95
Time (hr)									
Mean	1.91	3.43	5.61	7.68	2.49	4.1	5.95	8.37	152.77
MRE									
Mean*	1.363	1.371	1.376	1.394	1.373	1.352	1.361	1.358	1.355
*N* = 50									
$\hat{N}$									
RB	0.04	0.05	0.03	0.03	0.04	0	0.02	0	0.05
CV	12.79	12.6	12.37	12.88	13.22	12.58	12.64	12.67	13.15
Coverage	0.93	0.91	0.97	0.96	0.96	0.9	0.92	0.94	0.94
$\hat{\sigma }$									
RB	0.01	−0.09	−0.11	−0.1	0.04	−0.01	−0.02	−0.02	0.01
CV	10.42	7.68	7.34	7.43	10.94	8.83	8.57	8.58	9.41
Coverage	0.95	0.79	0.67	0.71	0.92	0.97	0.93	0.95	0.91
${\hat{p}}_{0}$									
RB	0.09	0.21	0.29	0.24	0.01	0.08	0.1	0.11	0.04
CV	23.11	21.62	21.21	21.65	25.23	23.04	22.74	22.84	24.06
Coverage	0.95	0.91	0.85	0.89	0.94	0.97	0.97	0.95	0.93
Time (hr)									
Mean	0.92	1.76	2.85	4.04	1.38	2.3	3.49	4.48	128.92
MRE									
Mean*	1.817	1.825	1.814	1.836	1.848	1.811	1.832	1.819	1.823

Notes

Results are presented for different AC evaluation window width and different detector evaluation window extensions (see Figure 5), expressed relative to *σ*. ^*^MRE are expressed ×10^−^^4^.

### 3.2 Wolverines

**Figure 8:**
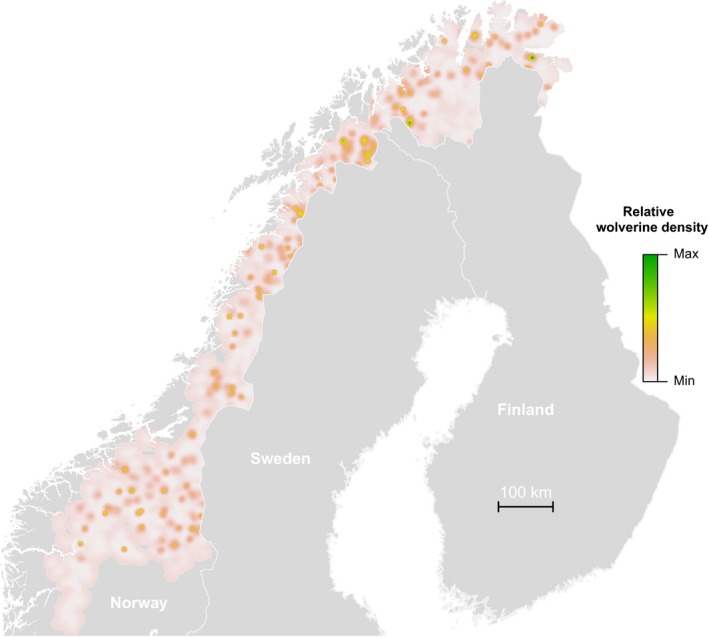
Map illustrating the relative density of female wolverines in 2012 in Norway estimated using a spatial capture–recapture (SCR) model applied to noninvasive genetic sampling data. National range‐wide mapping was made possible by the implementation of a local evaluation of the individual state‐space (LESS) in a SCR model during the model fitting which otherwise was not doable on a standard desktop machine. Due to the use of an overly simplistic model and likely violation of several model assumptions, the map is neither intended nor suitable for interpretation as an actionable result in terms of population management

Using the LESS approach, we were able to fit a SCR model to wolverine NGS data over the entire range of the species in Norway in less than 22 hr. The resulting map of relative density is shown in Figure 8 (Supporting Information Appendix S4). We reemphasize that this analysis is a proof of concept serving the sole purpose of demonstrating the feasibility of using LESS to enable analysis of very large state‐space problems using Bayesian methods.

## 4 DISCUSSION

Spatial capture–recapture methods are now widely used to investigate a range of important spatial ecological processes, and to inform conservation and management actions (Royle et al., 2018). The emergence of efficient inventory techniques such as noninvasive genetic sampling and remote camera trapping has resulted in a substantial increase in the spatial scales of some monitoring projects. The challenge that has emerged is computational in nature: finding tractable modeling procedures that allow SCR methods to be applied over large spatial domains and make inferences about population‐level processes. Our local evaluation method provides a solution that produces unbiased inferences, while vastly reducing the computational burden of enormous spatial models, and in doing so allows large‐scale Bayesian SCR models to be applied using a standard desktop in a fraction of the time required in the absence of LESS.

When using LESS in a Bayesian framework with data augmentation, our results suggest that there was virtually no bias in focal parameters when using an AC evaluation window width of 5*σ* and a detector evaluation window that extended by 2*σ* beyond the edge of the AC window (detector window width = 9*σ*)*.* A detector window of 9*σ *covers most of the half‐normal distribution defining the detection function (Figure 2). A 2*σ* extension of the detector window beyond the edges of the AC window corresponds to the general recommendations of habitat buffer size in SCR models (Royle et al., 2013). Using those settings in LESS, we measured up to 57 times gains in computing speed.

In SCR models, the computational burden increases rapidly with the number of detectors used (Milleret, Dupont, et al., 2018). The LESS approach, by using detector evaluation windows, reduces the number of detectors over which detection probability has to be estimated for each individual. In our simulated example, individual detection probability had to be estimated for each of the 2,500 detectors when a SCR model was fitted without LESS. However, using the LESS approach and our recommended AC and detector evaluation windows widths (AC: 5*σ*, detector: 9*σ*)*, *the maximum number of detectors for which individual detection probability had to be estimated was 324. Although the location of individual activity centers is also constrained to a smaller area using the AC evaluation window, such a reduction in the number of detectors used for each individual likely explains the major speed gains with LESS. This means that increasing the size of the problem (e.g., higher number of detector, larger spatial extent, or greater population size) will amplify the gains even further as demonstrated in the wolverine example. While we were unable to fit the wolverine SCR model for the entire Norwegian range of the species (>200,000 km^2^), the LESS approach allowed us to reduce the maximum number of detectors evaluated per individual from 17,266 to 500, and fit the model in less than 22 hr.

Implementing the LESS in a Bayesian SCR framework is straightforward, with proper indexing to define the windows requiring the most care (Figure 4). Although the half‐normal detection function represents a circular utilization of the space by individuals (Figure 2), we chose a square evaluation window for ease of indexing (Figure 3). This simplification did not seem to introduce additional bias.

Local evaluation is already possible in a maximum likelihood framework (oSCR, (Sutherland et al., 2017)) but remains rarely used or described (but see Sun, Fuller, Hare, & Hurst, 2017) and has yet to gain the attention it deserves. Conceptually, the approach used in oSCR is identical to our “2‐window” approach, but instead of using square evaluation windows, oSCR uses circular evaluation windows. First, a “trim” value defines the width of a buffer around individual detections that identifies where ACs can be located. Then, an additional buffer (double the width of the first buffer) is used to identify the detection ranges of all possible ACs for a given individual.

The SCR model with LESS that we used to demonstrate local evaluation did not include any covariates to account for individual/spatial heterogeneity in detection and space use. If detector‐specific covariates were to be included, similar indexing as on the detection function should be employed. Ultimately, the width of the windows should be specific to the study system; an optimal trade‐off between computation speed and reliability of prediction can be determined using sensitivity analysis. Customizable R code for simulation and model fitting is provided in the Supplementary information Appendix S1.

The use of data augmentation to estimate abundance in SCR is specific to Bayesian inferences (Royle et al., 2007, 2009). Although proven to be a robust tool to estimate abundance, dealing with data augmentation can be a challenge with more complex model formulation. For example, the correction that we applied to the inclusion parameter (*ψ_i_*) as a function of the size of available habitat for each individual is specific to augmented individuals. In cases where individuals show a high degree of spatial aggregation within the study area, density of augmented individuals should be larger than the maximum observed density, to ensure that enough AC windows are available for augmented individuals to estimate density in areas with high individual density. Additionally, in the case of open population SCR models (Bischof, Brøseth, et al., 2016), steps would have to be taken to deal with individual movement. Under an open population SCR model where a dispersal kernel is explicitly defined, or for nonstationary (no breaking) activity centers (Royle, Fuller, & Sutherland, 2016), there exists a spatial parameter similar to *σ* (dispersal *σ*) to redistribute potential ACs. The general rule of the LESS approach that restricts calculation where detection probability is >0 would also apply to where the relocation probability of an individual's AC is >0 (i.e., based on dispersal kernel function). As it stands, our approach does not accommodate for moving individuals because we defined static individual evaluation windows.

Ecological studies have an increasing need for methodological developments to analyze the ever‐increasing size of spatially explicit datasets and produce landscape, regional, and even global inferences about population state variables (Sutherland, Brambilla, Pedrini, & Tenan, 2016; Tenan, Brambilla, Pedrini, & Sutherland, 2017). The LESS approach presented here is a pragmatic redundancy reduction approach, as it avoids calculation that are unnecessary (i.e., where detection probability is close to 0). Although some studies have used such reduction to increase computational efficiency of spatial models (e.g., Latimer, Banerjee, Sang, Mosher, and Silander (2009), Gramacy (2016)), our approach could stimulate the development of more efficient model formulation that have a built‐in correlation structure.

## 5 CONCLUSION

SCR is a powerful (Royle et al., 2018), yet computationally demanding tool (Milleret, Dupont, et al., 2018). Incorporating LESS in SCR model opens the door to SCR application at large spatial scales, a prerequisite for comprehensive population‐level conservation and management plans (Bischof, Brøseth, et al., 2016). Because the computational burden associated with the spatial component of SCR models can be considerably reduced using LESS, it will also be possible to develop computationally efficient models that include relevant ecological processes (e.g., survival, recruitment, movement; Chandler, Hepinstall‐Cymerman, Merker, Abernathy‐Conners, & Cooper, 2018) at large spatial scales. Generally, we encourage practitioners to use local evaluation when performing SCR at large spatial scales, whether using Bayesian or maximum likelihood inference (Sutherland et al., 2017).

## CONFLICT OF INTEREST

None Declared.

## AUTHOR'S CONTRIBUTION

C.M, P.D and R.B developed the concept and methodology. R.B conceived the idea of the local evaluation in the Bayesian SCR model with data augmentation. H.B and Ø.F coordinated wolverine data extraction and preparation. C.M led the analysis with help from P.D and R.B, and C.B. to run the models. C.M led the writing with contributions of P.D, C.S, C.B and R.B. All authors contributed critically to drafts of the manuscript and gave final approval for publication.

## DATA ACCESSIBILITY

Wolverine data and associated R script are available on DRYAD entry https://doi.org/10.5061/dryad.42m96c8 (Milleret, Dupont, et al., 2018).
