# Supplementary material for: A local evaluation of the individual state‐space to scale up Bayesian spatial capture–recapture
Source: Ecol Evol. 2018 Dec 18;9(1):352–63. doi: 10.1002/ece3.4751 (PMC6342129; doi:10.1002/ece3.4751)

LOCAL EVALUATION OF THE INDIVIDUAL STATE-SPACE (LESS)- DATA SIMULATION & JAGS MODEL FITTING SCRIPT

This script performs a data set simulation and SCR modelling with the LESS using JAGS and rjags. All details about the procedure are provided in Milleret et al. (2018). A local evaluation of the individual state-space to scale up Bayesian spatial capture recapture. Ecology and Evolution

I.LOAD LIBRARIES AND SET WORKING DIRECTORY

```
rm(list=ls())
library(rgdal)
library(raster)
library(rgeos)
library(sp)
library(rjags)
library(jagsUI)
library(abind)
```

Rename the appendix S2 “SourceFunction.txt” to “SourceFunction.R” Set working directory where the SourceFunctions.R is located and source the file

```
setwd("YourWorkingdirectory")
source("SourceFunctions.R")
```

==== 1. LOAD SCR MODELS ====

```
      ### ==== 1.1 SCR  ====
sink("SCR.jags")
cat("model {
    ##------------------------------------------------------------
    ##-----------------------------------------##
    ##----------    AC PLACEMENT           ----##
    ##-----------------------------------------##
    for(i in 1:n.individuals){
    sxy[i,1] ~ dunif(0, x.max)
    sxy[i,2] ~ dunif(0, y.max)
    pOK[i] <- habitat.mx[trunc(sxy[i,2])+1, trunc(sxy[i,1])+1]
    OK[i] ~ dbern(pOK[i])
    }#i

    ##------------------------------------------------------------
    ##-----------------------------------------##
    ##---- DEMOGRAPHIC PROCESS ----------------##
    ##-----------------------------------------##
    psi ~ dunif(0,1)

    for (i in 1:n.individuals){
    z[i] ~ dbern(psi)
    }#i
```

```
    ##-------------------------------------------------------------
    ##-----------------------------------------##
    ##---- DETECTION PROCESS -----------------##
    ##-----------------------------------------##
    sigma ~ dunif(0,50)
    alpha <- -1/ (2*sigma*sigma)
    p0 ~ dunif(0,1)

    for (i in 1:n.individuals){
        for (j in 1:n.detectors){
             d2[i,j] <- pow(sxy[i,1] - detector.xy[j,1], 2) +
                        pow(sxy[i,2] - detector.xy[j,2], 2)
             p[i,j] <- p0 * exp(alpha * d2[i,j])
             y[i,j] ~ dbern(p[i,j]*z[i])
         }#j
    }#i

    ##-------------------------------------------------------------
    ##------------------------------------------##
    ##---------- DERIVED PARAMETERS ----------##
    ##------------------------------------------##
    N <- sum(z[])

    ##-------------------------------------------------------------
    }",fill = TRUE)
sink()

      ### ==== 1.2 SCR LESS  ====
sink("SCR-LESS.jags")
cat("model {
  ##---------------------------------------------------------------
  ##----------    AC PLACEMENT      ---##
  ##-----------------------------------##
  for(i in 1:n.individuals){
    sxy[i,1] ~ dunif(xy.bounds[i,1,1], xy.bounds[i,1,2])
    sxy[i,2] ~ dunif(xy.bounds[i,2,1], xy.bounds[i,2,2])
    pOK[i] <- habitat.mx[trunc(sxy[i,2])+1, trunc(sxy[i,1])+1]
    OK[i] ~ dbern(pOK[i])
  }#i
  ##---------------------------------------------------------------
  ##----- DEMOGRAPHIC PROCESS ----##
  ##------------------------------##
  psi0 ~ dunif(0,1)
  psi <- mean(psi1[])
  for (i in 1:n.individuals){
    psi1[i] <- 1-(1-psi0)^prop.habitat[i]
    z[i] ~ dbern(psi1[i])
  }#i
  ##---------------------------------------------------------------
  ##---------- DETECTION PROCESS------##
  ##----------------------------------##
  p0 ~ dunif(0,1)
  sigma ~ dunif(0,50)
```

```
  alpha <- -1/(2*sigma*sigma)
  #----- DETECTION PROCESS ------#
  for (i in 1:n.individuals){
    for (j in 1:n.detectors[i]){
      d2[i,j] <- pow(sxy[i,1] - detector.xy[detector.index[i,j], 1] ,2) +
                 pow(sxy[i,2] - detector.xy[detector.index[i,j], 2], 2)
      p[i,j] <- p0 * exp(alpha * d2[i,j])
      y[i,detector.index[i,j]] ~ dbern(p[i,j]*z[i])
    }#j
  }#i
  ##-------------------------------------------------------------
  ##---------- DERIVED PARAMETERS ----------##
  ##--------------------------------------##
  N <- sum(z[])
    }",fill = TRUE)
sink()
```

II.SET SIMULATION PARAMETERS

```
## Individuals
N <- 50                 ## Number of individuals to be simulated in the population

## Detections parameters of the halfnormal
sigma <- 2              ## sigma
p0 <- 0.07              ## p0

## Habitat
grid.size <- 50         ## Extent of the square defined as the habitat searched
                        ## (covered with detectors)
buffer <- sigma * 2     ## Habitat buffer size
resolution.habitat <- 1 ## Resolution of the habitat

## Detectors
detector.spacing <- 1   ## Dectector spacing

## Local evaluation
LESS <- TRUE    ## Whether local evaluation of the state-space (LESS) should be
                ## performed or not. If FALSE, be ready for heavy computing time!
Width.AC.Regions <- 5*sigma        ## Width of the AC evaluation window
Width.Detectors.Regions <- 9*sigma ## Width of the Detectors evaluation window
## Augmentation
augmentation <- 2       ## Number of times the data set should be
                        ## augmented.(when LESS=FALSE)
n.layers <- 5           ## Number of layers of augmented individuals
                        ## to be used with the LESS approach

## JAGS
ni <- 2000              ## Number of iterations
na <- 300               ## Number of iterations for the adaptive phase
nt <- 1                 ## Thining
nc <- 1                 ## Number of chains
nb <- 500               ## Burnin
```

III.SIMULATE DATA

```
  ### ==== 1. CREATE A SQUARE HABITAT WHERE DETECTORS WILL BE PLACED  ====

coords <- matrix(c(buffer          , buffer ,
                   grid.size+buffer, buffer ,
                   grid.size+buffer, grid.size+buffer,
                   buffer          , grid.size+buffer,
                   buffer          , buffer
                  ), ncol = 2, byrow = TRUE)

P1 <- Polygon(coords)
myStudyArea.poly <- SpatialPolygons(list(Polygons(list(P1), ID = "a")),
                                    proj4string=CRS(
"+proj=utm +zone=33 +datum=WGS84 +units=m +no_defs +ellps=WGS84 +towgs84=0,0,0"))


   ### ==== 2. CREATE A BUFFER AREA  ====
# This represents the total habitat
buffered.poly <- buffer(myStudyArea.poly, width = buffer)

      # PLOT TO CHECK
plot(buffered.poly)
plot(myStudyArea.poly,add=T)


   ### ==== 3. DEFINE SUITABLE HABITAT  ====
habitat.r <- raster(buffered.poly, res= resolution.habitat)
habitat.r <- rasterize(buffered.poly,habitat.r )

      # OBTAIN A MATRIX OF 0 AND 1 FOR NON-HABITAT/HABITAT
habitat.r[is.na(habitat.r[])] <- 0
habitat.mx <- as.matrix(habitat.r)

      # OBTAIN A MATRIX OF CELL ID FOR THE HABITAT RASTER
IDCells.r <- habitat.r
IDCells.r[] <- 1:ncell(IDCells.r)
IDCells.mx <- as.matrix(IDCells.r)

      # OBTAIN XY COORDINATES OF THE HABITAT
habitat.xy <- coordinates(habitat.r)
# trick so that the top left corner of the habitat has coordinates 0.5,0.5
habitat.xy[,2] <- abs(dim(habitat.r)[1]-habitat.xy[,2])

   ### ==== 4. SIMULATE RANDOM LOCATION OF AC CENTERS ====
simulated.ACS <- spsample(buffered.poly, n = N, type="random")
      # PLOT CHECK
points(simulated.ACS, pch=16, col="red")
```

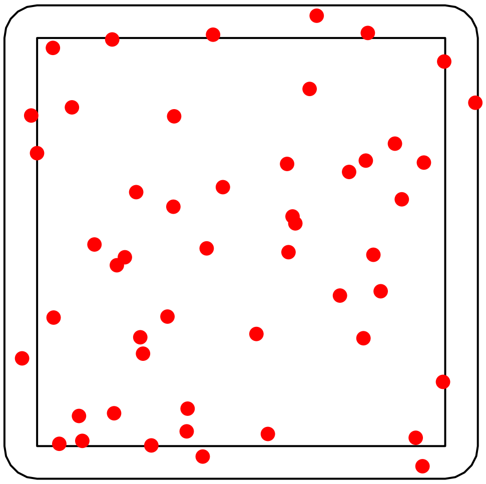

```
   ### ==== 5. CREATE DETECTORS ====
detectors.r <- raster(myStudyArea.poly, res= detector.spacing)
detectors.r <- rasterize(myStudyArea.poly, detectors.r)

      # OBTAIN XY COORDINATES OF DETECTORS
detectors.xy <- coordinates(detectors.r)
# trick so that the top left corner of the detectors has coordinates 0.5,0.5
detectors.xy[,2] <- abs(dim(habitat.r)[1]-detectors.xy[,2])
detectors.sp <- SpatialPoints(detectors.xy)
proj4string(detectors.sp) <- CRS(proj4string(simulated.ACS))

   ### ==== 6. SIMULATE DETECTIONS ====
      # OBTAIN DISTANCE MATRIX BETWEEN AC CENTERS AND DETECTORS
D <- gDistance(detectors.sp, simulated.ACS, byid=TRUE)

      # OBTAIN Y DETECTION MATRIX USING HALF NORMAL DETECTION FUNCTION (EQN 7 IN MAIN TEXT)
fixed.effects <- rep(log(p0), length(detectors.sp))
p0 <- exp(fixed.effects)
p <- p0 * exp(-D*D/(2*sigma*sigma))
y.all <- apply(p, c(1,2), function(x) rbinom(1, 1, x))

      # SLIM Y TO DETECTED INDIVIDUALS
detected <- apply(y.all, 1, max)>0
y.detect <- y.all[detected, ]
```

```
   ### ==== 7. STATE SPACE RESTRICTION (if SSRestriction==TRUE) AND DATA AUGMENTATION  ====
if(LESS==TRUE){
  myLESSIndices <- MakeLocalEvaluationIndexes ( y = y.detect
                                              , detector.xy = detectors.xy
                                              , habitat.xy = habitat.xy
                                              , Width.AC.Regions = Width.AC.Regions
                                              , Width.Detectors.Regions = Width.Detectors.Regions
                                              , min.y = NULL
                                              , max.y = NULL
                                              , min.x = NULL
                                              , max.x = NULL
                                              , n.layers = 5
                                              , habitat.mx = habitat.mx
                                              , IDCells.mx = IDCells.mx
                                              , plot.check = TRUE)



n.augmented <- myLESSIndices$n.individuals.augmented
y <- myLESSIndices$y.augmented

}else{
   # DATA AUGMENTATION IF SSRestriction==FALSE   ====
   n.augmented <- (augmentation*N) - nrow(y.detect)
   y.augmented <- matrix(0, nrow= n.augmented, ncol=ncol(y.detect))
   y <- rbind(y.detect, y.augmented)
}
```

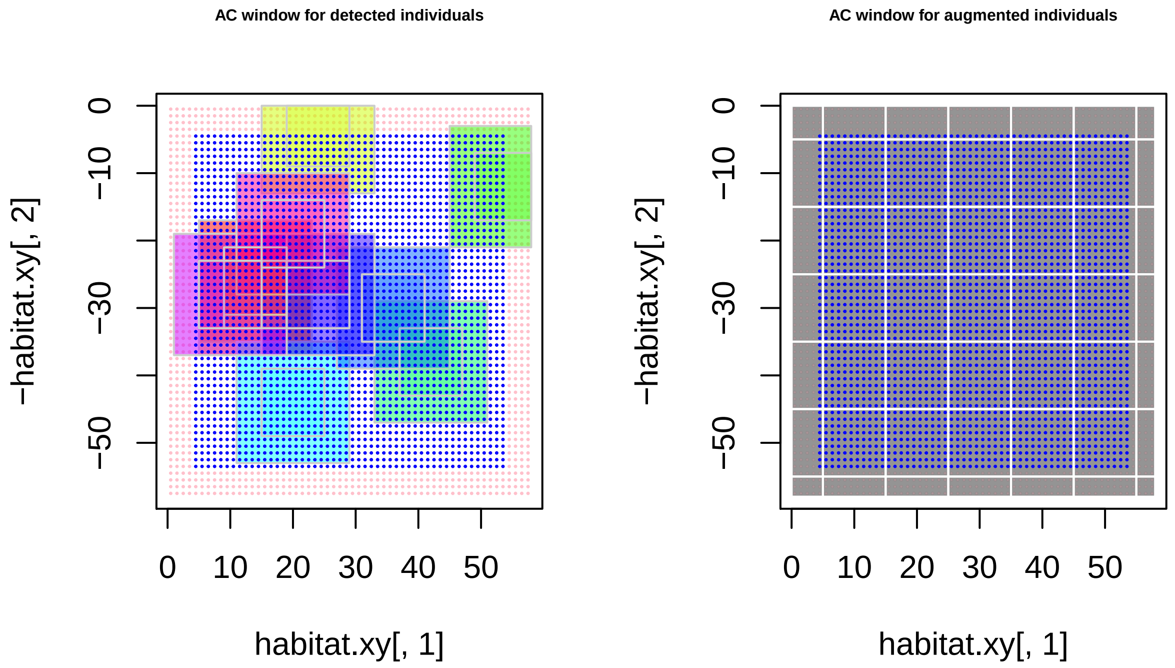

```
   ### ==== 8. INITIAL VALUES Z AND OK FOR THE ONES TRICK   ====
z <- rep(1,nrow(y.detect))
z <- c(z, rep(NA, n.augmented))
OK <- rep(1, dim(y)[1])
z.init <- ifelse(!is.na(z),NA,1)

# CREATE INITIAL VALUES
if(LESS==TRUE){
inits <- function(){list(
  "z" = z.init
 , "sxy" = MakeInitsXY( MovingWindows.AC.xy = myLESSIndices$MovingWindows.AC.xy
                      , habitat.mx = habitat.mx
                      , IDCells.mx = IDCells.mx
                      , habitat.xy = habitat.xy))}
  }else{
   inits <- function(){list( "z" = z.init)}
}


## -----------------------------------------------------------------------------------------
## ------ IV. RUN JAGS MODEL ---------------------------------------------------------------
## -----------------------------------------------------------------------------------------
   ### ==== 1. PREPARE DATA FOR JAGS   ====
   # DATA FOR JAGS
myJagsInput <- list(
  z = z ## State vector
, y = y ## Detection array
, n.individuals = dim(y)[1] ## Number of individuals (augmented)
, detector.xy = detectors.xy ## Detector xy positions
, detector.index = myLESSIndices$detector.index  ## Individual detector indices
, habitat.mx = habitat.mx  ## Suitable habitat matrix
, xy.bounds = myLESSIndices$xy.bounds.AC ## Moving AC Window boundaries
, n.detectors = myLESSIndices$n.detectors ## Number of detectors per moving window
, prop.habitat = myLESSIndices$prop.habitat.window ## Proportion of suitable
                                                   ## habitat cells within
                                                   ## the AC regions
, OK = rep(1, length(z))) ## Ones' trick vector

parms <- c("N", "sigma", "p0")

if(LESS==TRUE){
   model.file <- "SCR-LESS.jags"
}else{
   model.file <- "SCR.jags" # to use if a standard SCR is to be computed.
                            # WARNINGS!! heavy computing time!
}


   ### ==== 2. RUN JAGS MODEL USING RJAGS  ====
#       # ADAPTIVE PHASE
# temp.model <- jags.model(  file = model.file
#                          , data = my.jags.input
#                          , inits = list("z" = z.init)
```

```r
#                             , n.chains = nc
#                             , n.adapt = na)
#
#       # GENERATE POSTERIOR SAMPLES
# jagsoutput <- coda.samples( model = temp.model
#                              , variable.names = parameters
#                              , n.iter = ni
#                              , thin = nt
# )
# MERGE THE CHAINS
# jags.output <- do.call(rbind,jagsoutput)

### ==== 3. RUN MODELS WITH JAGSUI TO BE ABLE TO PARRELLIZE IT  ====
jagsoutput <- jagsUI::jags( data = myJagsInput
                             , inits = inits
                             , parameters.to.save = parms
                             , model.file = model.file
                             , n.chains = nc
                             , n.adapt = na
                             , n.iter = ni
                             , n.burnin = nb
                             , n.thin = nt
                             , parallel = TRUE
                             , DIC = FALSE
                             , bugs.format = TRUE)
```

```
##
## Processing function input.......
##
## Done.
##
## Compiling model graph
##    Resolving undeclared variables
##    Allocating nodes
## Graph information:
##    Observed stochastic nodes: 51083
##    Unobserved stochastic nodes: 816
##    Total graph size: 1247372
##
## Initializing model
##
## Adaptive phase, 300 iterations x 1 chains
## If no progress bar appears JAGS has decided not to adapt
##
##
##  Burn-in phase, 500 iterations x 1 chains
##
##
## Sampling from joint posterior, 1500 iterations x 1 chains
##
##
## Calculating statistics.......
```

```
## Warning in process.output(samples, DIC = DIC, codaOnly, verbose = verbose):
```

```
## At least one Rhat value could not be calculated.
##
## Done.
```

```r
## ----------------------------------------------------------------------------------------
## ------ V. PLOT RESULTS JAGS MODEL ---------------------------------------------------
## ----------------------------------------------------------------------------------------
   ### ==== 1. COMPARE ESTIMATED VS SIMULATED VALUES    ====

par(mfrow=c(1,3),mar=c(3,3,2,2))
# SIGMA
plot(density(jagsoutput$sims.list$sigma), main="Sigma")
abline(v=sigma, col="red")

# p0
plot(density(jagsoutput$sims.list$p0), main="p0")
abline(v=p0, col="red")

# N
plot(density(jagsoutput$sims.list$N), main="POP.SIZE")
abline(v=N, col="red")
```

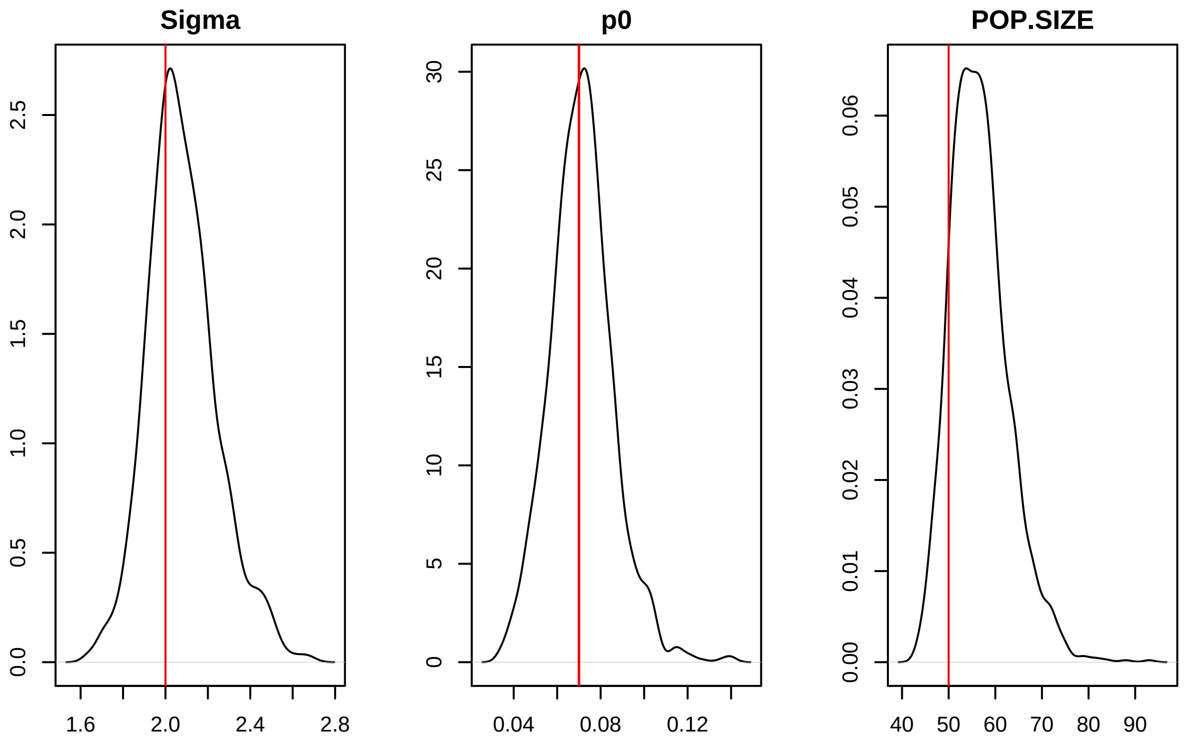

Supplement: Supplementary file 1 [file ECE3-9-352-s001.pdf]
