# Supplementary material for: A local evaluation of the individual state‐space to scale up Bayesian spatial capture–recapture
Source: Ecol Evol. 2018 Dec 18;9(1):352–63. doi: 10.1002/ece3.4751 (PMC6342129; doi:10.1002/ece3.4751)
Supplement: Supplementary file 2 [file ECE3-9-352-s002.docx]

## ---- R FUNCTIONS NECESSARY TO RUN THE SCR MODEL WITH LOCAL EVALUATION OF THE INDIVIDUAL STATE-SPACE (LESS)- DATA SIMULATION & JAGS### Milleret et al. 2018. A local evaluation of the individual state-space to scale up Bayesian spatial capture recapture. Ecology and Evolution

## ==== I. MakeLocalEvaluationIndexes.R ====

#' @title MakeLocalEvaluationIndexes

#'

#' @description

#' \code{MakeLocalEvaluationIndexes} returns a list object with all objects containing indices to perform a local evaluation of the state space in SCR

#' @param y \code{Numeric } detection history matrix, with individuals in rows and detectors in columns (the matrix should only contain indidividuals with >0 detection)

#' @param detector.xy \code{Numeric} Matrix with detector coordinates (x and y in columns).

#' @param habitat.xy \code{Numeric} Matrix with habitat coordinates (raster cell centroid coordinates) (x and y in columns).

#' @param habitat.mx \code{Matrix} Habitat matrix with 1: habitat and 0 : non-habitat

#' @param Width.AC.Regions \code{Numeric} Width of the sides of the square extent window that defines the boundaries of the individual AC regions

#' @param Width.Detectors.Regions \code{Numeric} Width of the sides of the square extent window that defines the boundaries of the individual detector regions

#' @param n.layers \code{Numeric} with the number of layers of augmented individuals to be added.

#' @param IDCells.mx \code{Matrix} with id of the cells of (habitat.mx) that matches with habitat.xy

#' @param plot.check A \code{logical} for whether (\code{TRUE}) or not (\code{FALSE}) plots are to be generated.

#' @param centroid.method \code{string} whether individual centroids of detected individuals should be defined annually ("yearly") or kept constant ("overall")

#' @param min.y A \code{Numeric} to force regions of augmented layers to be located in particular location of the study area.

#' If min.y is defined, the lower corner of the lower region within the study area will have the defined min.y coordinates. (The same aplies to max.y, min.x, max.x)

MakeLocalEvaluationIndexes <- function( y

, detector.xy

, habitat.xy

, habitat.mx

, Width.AC.Regions

, Width.Detectors.Regions

, n.layers

, IDCells.mx

, plot.check=TRUE

, min.y = NULL

, max.y = NULL

, min.x = NULL

, max.x = NULL){

# NUMBER OF DETECTED INDVIDUALS

n.individuals.detected <- dim(y)[1]

if((Width.AC.Regions %% 2)==1){

Width.AC.Regions <- Width.AC.Regions+1

print(paste("WARNINGS!!! Width.AC.Regions is not an even value and has been rounded to", Width.AC.Regions, " so it matches the habitat"))

}

##==== I. OBTAIN THE CENTER OF DETECTIONS FOR DETECTED INDIVIDUALS ====

id.detectors <- lapply(1:n.individuals.detected, function(x){which(y[x, ] > 0)})

xy.center.detected <- do.call(rbind, lapply(id.detectors, function(x){

x <- rbind(detector.xy[x,], c(NA,NA))

apply(x, 2, function(y) mean(y, na.rm=TRUE))})

)

##==== II. OBTAIN THE CENTER REGIONS FOR AUGMENTED INDIVIDUALS ====

##---- 1. ADJUST START AND END OF THE PLACEMENT OF THE AC REGIONS

if(is.null(max.y)){max.y <- round(max(habitat.xy[,2])) + Width.AC.Regions }

if(is.null(max.x)){max.x <- round(max(habitat.xy[,1])) + Width.AC.Regions }

if(is.null(min.x)){min.x <- round(min(habitat.xy[,1])) - Width.AC.Regions }

if(is.null(min.y)){min.y <- round(min(habitat.xy[,2])) - Width.AC.Regions }

## ---- 2. DEFINE AC CENTER OF AUGMENTED INDIVIDUALS ----

x.seq <- seq(min.x, max.x, by = Width.AC.Regions)

y.seq <- seq(min.y, max.y, by = Width.AC.Regions)

aug0.xy <- as.data.frame(expand.grid(x.seq, y.seq))

# plot(aug0.xy[,2] ~ aug0.xy[,1], pch=16, cex=0.4)

# points(habitat.xy[,2] ~ habitat.xy[,1], pch=16, col="red", cex=0.4)

# points(detector.xy[,2] ~ detector.xy[,1], pch=16, col="blue", cex=0.4)

# points(aug0.xy[,2] ~ aug0.xy[,1], pch=16, cex=0.4)

##---- 3. ADD NUMBER OF LAYERS OF AUGMENTED INDIVIDUALS

aug.xy <- do.call(rbind, lapply(1:n.layers, function(x) aug0.xy))

## N INDIVIDUALS

n.individuals.augmented <- dim(aug.xy)[1]

n.individuals <- n.individuals.detected + n.individuals.augmented

##==== III. MERGE AND CREATE THE CENTER REGIONS FOR ALL INDIVIDUALS ====

## ---- 1. FOR AC REGIONS ----

xy.center <- abind(round(xy.center.detected), aug.xy, along = 1)

AC.regions.xy <- abind(xy.center, xy.center - round(Width.AC.Regions/2), round(xy.center + Width.AC.Regions/2), along = 2)

dimnames(AC.regions.xy) <- list(1:n.individuals, c("x", "y", "lower.x", "lower.y", "upper.x", "upper.y"))

## ---- 2. FOR DETECTORS REGIONS ----

DETECTOR.regions.xy <- AC.regions.xy

diff.windows <- (Width.Detectors.Regions-Width.AC.Regions)/2

# extend REGIONS FOR detectors

DETECTOR.regions.xy[,c("lower.x","lower.y")] <- DETECTOR.regions.xy[,c("lower.x","lower.y")] - diff.windows

DETECTOR.regions.xy[,c("upper.x","upper.y")] <- DETECTOR.regions.xy[,c("upper.x","upper.y")] + diff.windows

## ---- 3. CUT AC AND DETECTOR REGIONS TO HABITAT EXTENT ----

xmin <- min(floor(habitat.xy[,1]))

ymin <- min(floor(habitat.xy[,2]))

xmax <- max(ceiling(habitat.xy[,1]))

ymax <- max(ceiling(habitat.xy[,2]))

#DETECTORS

DETECTOR.regions.xy[DETECTOR.regions.xy[ ,"lower.x"] < xmin, "lower.x"] <- xmin

DETECTOR.regions.xy[DETECTOR.regions.xy[ ,"lower.y"] < ymin, "lower.y"] <- ymin

DETECTOR.regions.xy[DETECTOR.regions.xy[ ,"upper.x"] > xmax, "upper.x"] <- xmax

DETECTOR.regions.xy[DETECTOR.regions.xy[ ,"upper.y"] > ymax, "upper.y"] <- ymax

#AC

AC.regions.xy[AC.regions.xy[ ,"lower.x"] < xmin, "lower.x"] <- xmin

AC.regions.xy[AC.regions.xy[ ,"lower.y"] < ymin, "lower.y"] <- ymin

AC.regions.xy[AC.regions.xy[ ,"upper.x"] > xmax, "upper.x"] <- xmax

AC.regions.xy[AC.regions.xy[ ,"upper.y"] > ymax, "upper.y"] <- ymax

## ---- 4. CREATE XY.BOUNDS DETECTORS AND AC FOR JAGS ----

#DETECTORS

xy.bounds.detectors <- array(NA, c(n.individuals, 2, 2))

xy.bounds.detectors[ , ,1] <- DETECTOR.regions.xy[,c("lower.x", "lower.y")]

xy.bounds.detectors[ , ,2] <- DETECTOR.regions.xy[,c("upper.x", "upper.y")]

#AC

xy.bounds.AC <- array(NA, c(n.individuals, 2, 2))

xy.bounds.AC[ , ,1] <- AC.regions.xy[,c("lower.x", "lower.y")]

xy.bounds.AC[ , ,2] <- AC.regions.xy[,c("upper.x", "upper.y")]

##==== IV.IDENTIFY DETECTORS IN DETECTOR REGIONS ====

# DETECTORS LIST PER REGION

detector.index.list <- lapply(1:n.individuals, function(x){which( detector.xy[ ,1] >= DETECTOR.regions.xy[x,3]

& detector.xy[ ,2] >= DETECTOR.regions.xy[x,4]

& detector.xy[ ,1] <= DETECTOR.regions.xy[x,5]

& detector.xy[ ,2] <= DETECTOR.regions.xy[x,6])})

n.detectors.id <- unlist(lapply(detector.index.list, length))

max.n.detectors <- max(n.detectors.id)

detector.index <- do.call(rbind, lapply(detector.index.list, function(x) c(x, rep(NA, max.n.detectors - length(x)))))

# COUNT NUMBER OF DETECTORS

n.detectors.id <- unlist(lapply(detector.index.list, length))

##==== V.REMOVE REGIONS WITH NO DETECTORS ====

regions.with.detectors <- which(n.detectors.id!=0)

## SUBSET THE OBJECTS

detector.index <- detector.index[regions.with.detectors,]

n.detectors.id <- n.detectors.id[regions.with.detectors]

xy.bounds.AC <- xy.bounds.AC[regions.with.detectors,,]

AC.regions.xy <- AC.regions.xy[regions.with.detectors,]

DETECTOR.regions.xy <- DETECTOR.regions.xy[regions.with.detectors,]

xy.bounds.detectors <- xy.bounds.detectors[regions.with.detectors,,]

# UPDATE N.INDIVIDUALS

n.individuals <- dim(xy.bounds.detectors)[1]

##==== VI.OBTAIN PERCENT OF HABITAT IN EACH MOVING WINDOW ====

# MAKE XY BOUNDS MATCHING WITH MATRIX OBJECTS

dim.mx <- xy.bounds.AC

dim.mx[,1,1] <- dim.mx[,1,1] +1

dim.mx[,2,1] <- dim.mx[,2,1] +1

# Obtain number of habitat cells covered by window size

size.habitat.window <- abs(x.seq[1]-x.seq[2])

# Obtain percent of habitat cells covered by window size

prop.habitat.window <- rep(0,dim(dim.mx)[1])

for(i in 1:dim(dim.mx)[1]){

# define X an Y extent of the moving window

min.x <- dim.mx[i,1,1]

max.x <- dim.mx[i,1,2]

min.y <- dim.mx[i,2,1]

max.y <- dim.mx[i,2,2]

test <- try(habitat.mx[c(min.y:max.y), c(min.x:max.x)], silent=TRUE)## remove the window with a detector but no habitat

if(class(test) %in% 'try-error'){next}

window.mx.ind <- as.matrix(habitat.mx[c(min.y:max.y), c(min.x:max.x)])

window.ID.ind <- as.matrix(IDCells.mx[c(min.y:max.y), c(min.x:max.x)])

if(sum(window.mx.ind)==0){

next

}

prop.habitat.window[i] <- length(window.ID.ind[window.mx.ind==1])/(size.habitat.window^2)

}

##==== VII.REMOVE REGIONS WITH NO HABITAT ====

regions.with.habitat <- which(prop.habitat.window!=0)

## SUBSET THE OBJECTS

detector.index <- detector.index[regions.with.habitat,]

n.detectors.id <- n.detectors.id[regions.with.habitat]

xy.bounds.AC <- xy.bounds.AC[regions.with.habitat,,]

AC.regions.xy <- AC.regions.xy[regions.with.habitat,]

DETECTOR.regions.xy <- DETECTOR.regions.xy[regions.with.habitat,]

xy.bounds.detectors <- xy.bounds.detectors[regions.with.habitat,,]

prop.habitat.window <- prop.habitat.window [regions.with.habitat]

# UPDATE N.INDIVIDUALS

n.individuals <- dim(xy.bounds.detectors)[1]

n.individuals.augmented <- n.individuals -n.individuals.detected

##==== VIII.CREATE AN AUGMENTED Y ====

y.augm <- abind(y, array(0, c(n.individuals.augmented, dim(y)[2])), along = 1)

## PLOT CHECK

if(plot.check){

par(mfrow=c(1,2))

ID.plots <- sample(1:n.individuals.detected, 10)

cols <- rainbow(length(ID.plots))

plot(-habitat.xy[,2]~ habitat.xy[,1], col = "pink", pch = 19, cex = 0.1, main= "AC window for detected individuals")

lapply(ID.plots, function(i){

xv <- c(DETECTOR.regions.xy[i,"upper.x"], DETECTOR.regions.xy[i,"lower.x"], DETECTOR.regions.xy[i,"lower.x"], DETECTOR.regions.xy[i,"upper.x"])

yv <- c(-DETECTOR.regions.xy[i,"upper.y"], -DETECTOR.regions.xy[i,"upper.y"], -DETECTOR.regions.xy[i,"lower.y"],-DETECTOR.regions.xy[i,"lower.y"])

polygon(xv, yv, border = "gray80", col = adjustcolor(cols[which(ID.plots==i)], alpha.f = 0.5))

})

lapply(ID.plots, function(i){

xv <- c(AC.regions.xy[i,"upper.x"], AC.regions.xy[i,"lower.x"], AC.regions.xy[i,"lower.x"], AC.regions.xy[i,"upper.x"])

yv <- c(-AC.regions.xy[i,"upper.y"], -AC.regions.xy[i,"upper.y"], -AC.regions.xy[i,"lower.y"],-AC.regions.xy[i,"lower.y"])

polygon(xv, yv, border = "gray80", col = adjustcolor(cols[which(ID.plots==i)], alpha.f = 0.2))

})

points(-detector.xy[,2]~detector.xy[,1] , col = "blue", pch = 19, cex = 0.1)

# ----- AUGMENTED

plot(-habitat.xy[,2]~ habitat.xy[,1], col = "pink", pch = 19, cex = 0.1, main= "AC window for augmented individuals")

lapply((n.individuals.detected+1):n.individuals, function(i){

xv <- c(AC.regions.xy[i,"upper.x"], AC.regions.xy[i,"lower.x"], AC.regions.xy[i,"lower.x"], AC.regions.xy[i,"upper.x"])

yv <- c(0-AC.regions.xy[i,"upper.y"], 0-AC.regions.xy[i,"upper.y"], 0-AC.regions.xy[i,"lower.y"], 0-AC.regions.xy[i,"lower.y"])

polygon(xv, yv, border = "white", col = grey(0.5, alpha = 0.3))

})

points(-habitat.xy[,2]~ habitat.xy[,1], col = adjustcolor("pink",alpha.f = 0.2), pch = 19, cex = 0.01, main= "AC window for detected individuals")

points(-detector.xy[,2]~detector.xy[,1] , col = "blue", pch = 19, cex = 0.1)

}

# ---- STEP 11: OUTPUT -----

out <- list( y.augmented = y.augm

, n.detectors = n.detectors.id

, detector.index = detector.index

, xy.bounds.AC = xy.bounds.AC

, xy.bounds.detectors = xy.bounds.detectors

, MovingWindows.detectors.xy = DETECTOR.regions.xy

, MovingWindows.AC.xy = AC.regions.xy

, n.individuals = n.individuals

, n.individuals.detected = n.individuals.detected

, n.individuals.augmented = n.individuals.augmented

, prop.habitat.window = prop.habitat.window)

return(out)

}

## ==== II. MakeInitsXY.R ====

#' @title MakeInitsXY

#'

#' @description

#' \code{MakeInitsXY} returns initial values to perform a local evaluation of the state space in SCR

#' @param MovingWindows.AC.xy \code{Matrix} the MovingWindows.AC.xy object returned from (MakeLocalEvaluationIndexes)

#' @param IDCells.mx \code{Matrix} Matrix ID of the habitat cells

#' @param habitat.xy \code{Numeric} Matrix with habitat coordinates (raster cell centroid coordinates) (x and y in columns).

#' @param habitat.mx \code{Matrix} Habitat matrix with 1: habitat and 0 : non-habitat

MakeInitsXY <- function( MovingWindows.AC.xy = myFragIndices$MovingWindows.AC.xy

,

habitat.mx = habitat.mx

,

IDCells.mx = IDCells.mx

,

habitat.xy = habitat.xy

){

sxy <- matrix(NA, ncol=2, nrow=dim(MovingWindows.AC.xy)[1])

for(i in 1:dim(MovingWindows.AC.xy)[1]){

IDS.habitat.mx <-habitat.mx[c((MovingWindows.AC.xy[i,"lower.y"]+1) : MovingWindows.AC.xy[i,"upper.y"]),

c((MovingWindows.AC.xy[i,"lower.x"]+1) : MovingWindows.AC.xy[i,"upper.x"] ) ]

IDS.cells <- IDCells.mx[c((MovingWindows.AC.xy[i,"lower.y"]+1) : MovingWindows.AC.xy[i,"upper.y"]),

c((MovingWindows.AC.xy[i,"lower.x"]+1) : MovingWindows.AC.xy[i,"upper.x"] ) ]

ID.Cells.Habitat <- IDS.cells[which(IDS.habitat.mx==1, arr.ind = T)]

if(length(ID.Cells.Habitat)==1){

sxy[i,] <- habitat.xy[ID.Cells.Habitat,]

}else{

sxy[i,] <- habitat.xy[sample(ID.Cells.Habitat,1),]

}

}

return(sxy)

}
