# Supplementary material for: A local evaluation of the individual state‐space to scale up Bayesian spatial capture–recapture
Source: Ecol Evol. 2018 Dec 18;9(1):352–63. doi: 10.1002/ece3.4751 (PMC6342129; doi:10.1002/ece3.4751)
Supplement: Supplementary file 3 [file ECE3-9-352-s003.docx]

**S3: Supplementary Table**

**Table S3.1**. Description of priors used in the JAGS SCR models. We provided initial values for *z* as a vector of 1 for detected individuals and *NA* for augmented individuals. We also provided initial *sxy* values as a matrix with the average coordinates of detections for detected individuals and a random location within AC evaluation windows for augmented individuals.

| **Parameter** | **Description** | **Priors** |
| --- | --- | --- |
| *σ* | Scale-parameter of the detection function | dunif(0, 100) |
| $\lambda_{0}$ | Expected number of detections at the AC | dunif(0, 15) |
| *ψ* | Inclusion probability | dunif(0, 1) |
| *Sx* | x coordinates of activity center locations | dunif(0, x.max) |
| *Sy* | y coordinates of activity center locations | dunif(0, y.max) |
| *Ψ_0_* | Weighed psi according to the proportion of suitable habitat (specific to SCR with LESS) | dunif(0, 1) |
