# Supplementary material for: A local evaluation of the individual state‐space to scale up Bayesian spatial capture–recapture
Source: Ecol Evol. 2018 Dec 18;9(1):352–63. doi: 10.1002/ece3.4751 (PMC6342129; doi:10.1002/ece3.4751)

Supporting information S4. Milleret et al. A local evaluation of the individual state-space to scale up Bayesian spatial capture recapture

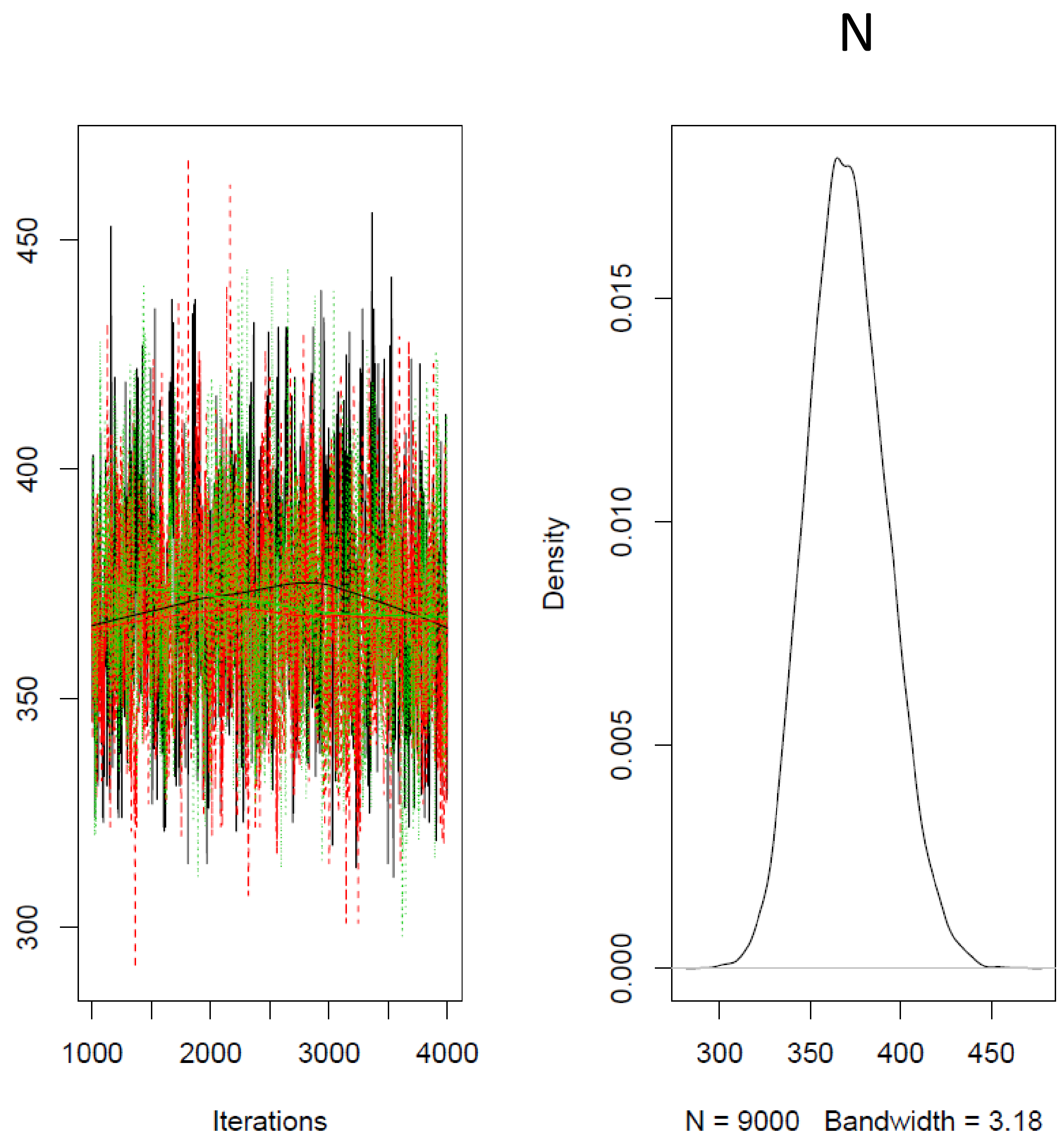

Figure S4-1. Trace plots of the N estimates obtained using the wolverines data collected in 2012 in Norway analyzed with a SCR model with a local evaluation of the state-space. Due to the use of an overly simplistic model and likely violation of several model assumptions, the estimates are neither intended nor suitable for interpretation as an actionable result in terms of population management. Using the LESS approach, the total graph size of the wolverines JAGS model JAGS was reduced by 5351156 nodes.

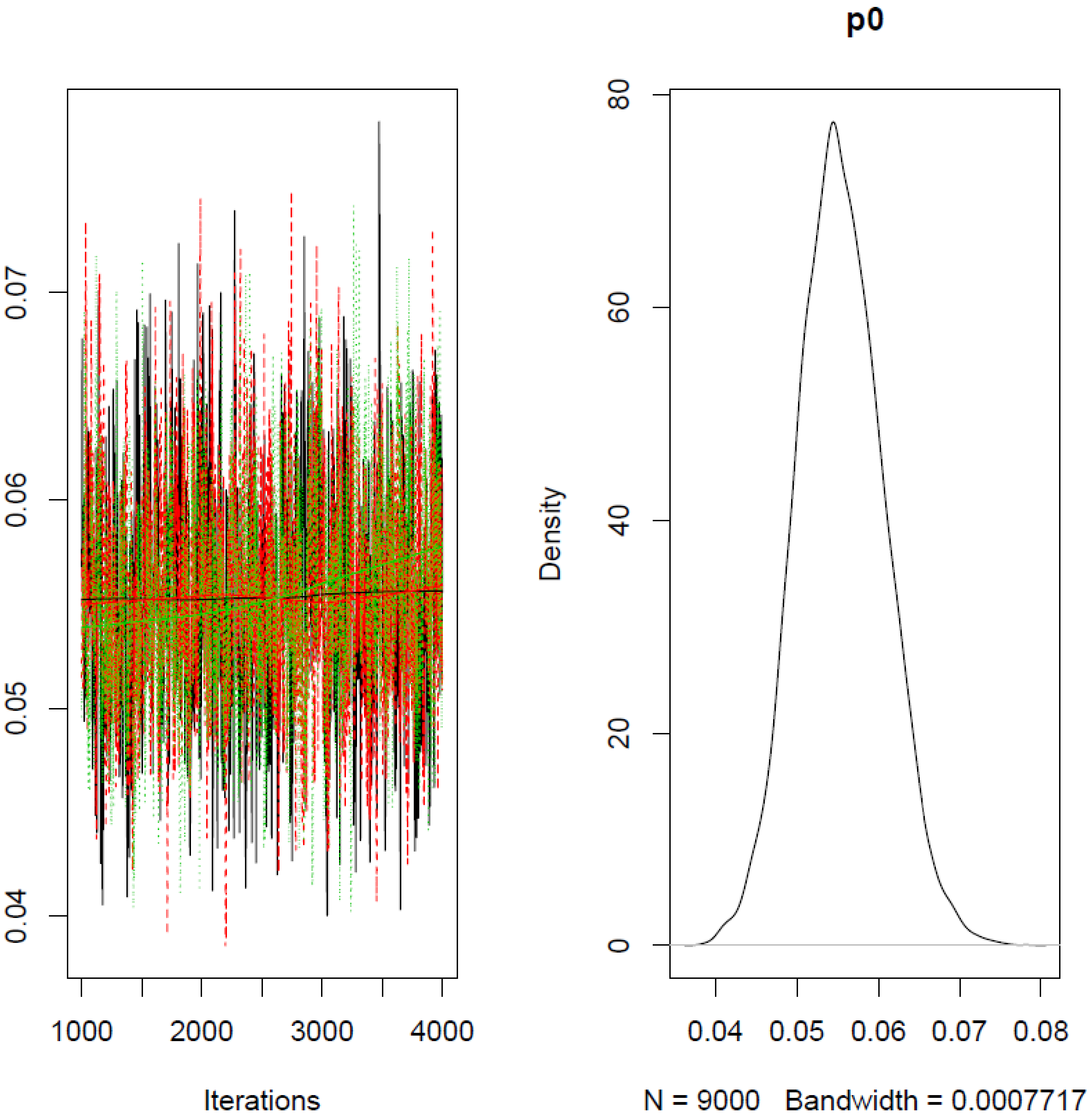

Figure S4-2. Trace plots of the p0 estimates obtained using the wolverines data collected in 2012 in Norway analyzed with a SCR model with a local evaluation of the state-space. Due to the use of an overly simplistic model and likely violation of several model assumptions, the estimates are neither intended nor suitable for interpretation as an actionable result in terms of population management. Using the LESS approach, the total graph size of the wolverines JAGS model JAGS was reduced by 5351156 nodes.

Figure S4-3. Trace plots of the σ estimates (in kilometers) obtained using the wolverines data collected in 2012 in Norway analyzed with a SCR model with a local evaluation of the state-space. Due to the use of an overly simplistic model and likely violation of several model assumptions, the estimates are neither intended nor suitable for interpretation as an actionable result in terms of population management. Using the LESS approach, the total graph size of the wolverines JAGS model JAGS was reduced by 5351156 nodes.

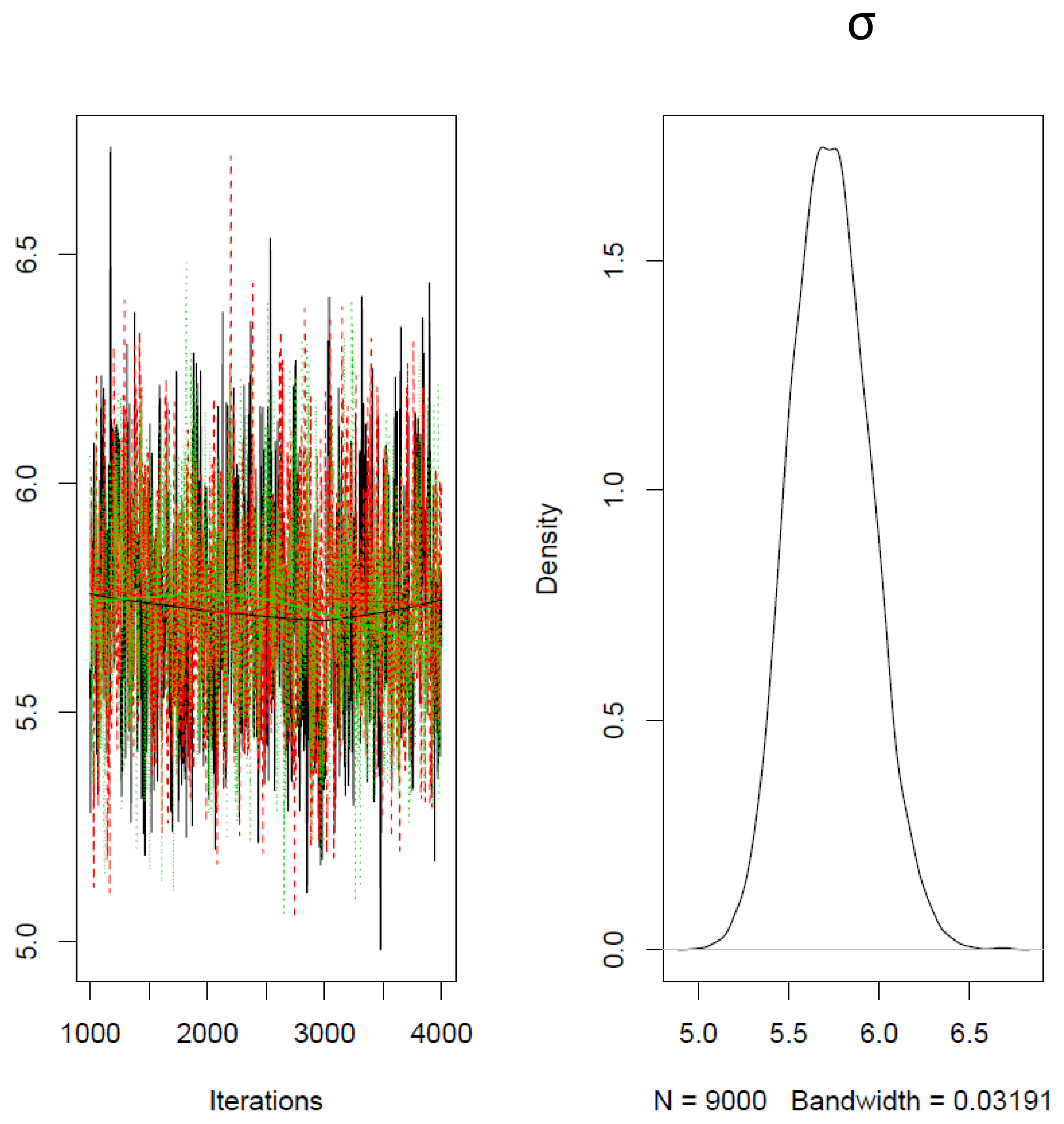

Supplement: Supplementary file 4 [file ECE3-9-352-s004.pdf]
